# Studying neural responses for multi-component economic choices in human and non-human primates using concept-based behavioral choice experiments

**DOI:** 10.1016/j.xpro.2023.102296

**Published:** 2023-06-08

**Authors:** Alexandre Pastor-Bernier, Konstantin Volkmann, Leo Chi U Seak, Arkadiusz Stasiak, Charles R. Plott, Wolfram Schultz

**Affiliations:** 1 Department of Physiology, Development and Neuroscience, University of Cambridge, Cambridge CB2 3DY, UK; 2 Division of Humanities and Social Sciences, California Institute of Technology, Pasadena, CA 91125, USA

**Keywords:** Model Organisms, Neuroscience, Cognitive Neuroscience, Behavior, Systems biology

## Abstract

Realistic, everyday rewards contain multiple components, such as taste and size. However, our reward valuations and the associated neural reward signals are single dimensional (vector to scalar transformation). Here, we present a protocol to identify these single-dimensional neural responses for multi-component choice options in humans and monkeys using concept-based behavioral choice experiments. We describe the use of stringent economic concepts to develop and implement behavioral tasks. We detail regional neuroimaging in humans and fine-grained neurophysiology in monkeys and describe approaches for data analysis.

For complete details on the use and execution of this protocol, please refer to our work on humans Seak et al.^1^ and Pastor-Bernier et al.^2^ and monkeys Pastor-Bernier et al. ^3^, Pastor-Bernier et al.^4^, and Pastor-Bernier et al.^5^.

## Highlights

• Design a behavioral task using proven concepts to study associated neural responses
• Apply the same task for both human neuroimaging and monkey neurophysiology
• Assess brain structures involved in economic decision-making from both species
• Provide adequate controls to demonstrate meaningful choices

## Abstract (editor highlights)

Publisher’s note: Undertaking any experimental protocol requires adherence to local institutional guidelines for laboratory safety and ethics.

## Graphical abstract

**Figure:**
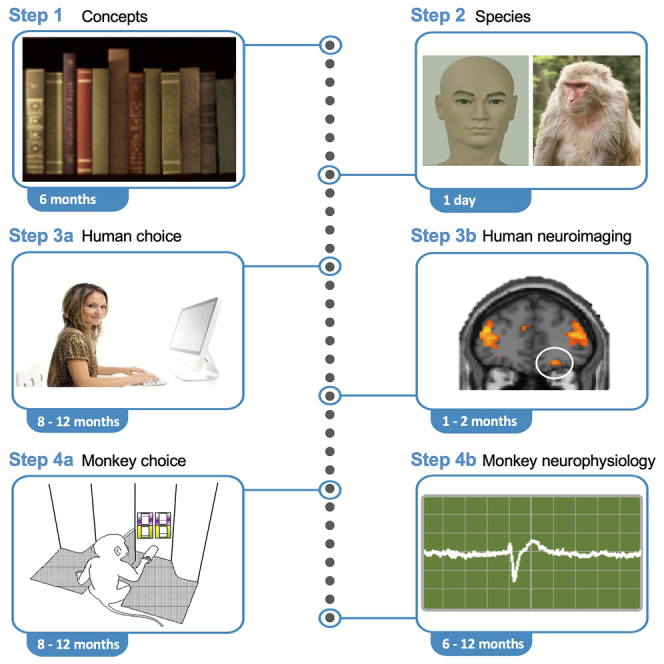

## Before you begin

The protocol provides an extensive guide for designing, running and evaluating a series of combined behavioral, neuroimaging and neurophysiological experiments on choices of multi-component options in humans and macaque monkeys. The protocol starts by implementing proven concepts of economic choice theory for multi-component options (Revealed Preference Theory). Following adequate behavioral data collection, the neural part of the experiment investigates, separately in humans and monkeys, how neural signals that vary only in one dimension can represent the two-component rewards. The protocol is laid-out general enough to be applicable to many choice scenarios and their neural investigations.

### Concepts

**Timing: 6 months**

Economic concepts developed, scrutinized and proven over decades of experimental and theoretical work provide a sound basis for designing behavioral and neural studies of reward and decision-making. This approach should allow the new results to be integrated into existing theories, update these theories, and possibly reduce them to an explanatory minimum (theory reduction). Such an approach using animal learning theory has allowed us to use Pavlovian and temporal difference reinforcement learning concepts for understanding the dopamine reward prediction error signal and integrate the observed neuronal mechanisms into reinforcement learning theory.^6^^,^^7^ We are now following a similar rationale with economic choice theory to investigate a crucial aspect of reward processing, the multi-component nature of rewards and their representation by single-dimensional neural reward signals. We suggest to advance in the following steps.

1. Consider fundamental concepts of reward and economic choice.^8^
  a. Define reward: any event, substance, object or biological organism that elicits approach behavior, consummatory behavior and learning in a biological human or animal agent. Examples are food, liquids and consumer goods.
  b. Define reinforcement: the consequence of an action that increases the intensity or probability of that action. Rewards are positive reinforcers, as they increase the behavior that resulted in the reward.
  c. Define subjective reward value: the goodness of a reward for a biological organism, formally known as utility.
  d. Define economic choice: selecting an option from a set of options (‘option set’).
  e. Define discrete choice:
    i. Restrict option sets to two or three options. While being more representative of everyday life, the analysis of larger option sets becomes more complicated.
    ii. Make option sets collectively exhaustive, such that a given option set contains all available options.
    iii. Make all options in a given option set distinct and separable.
    iv. Make all options in a given option set mutually exclusive, such that an agent can obtain only one option.
2. Define the designed test variables.
  a. In a given option set, set amounts of rewards independently of each other.
  b. Present all options of a given option set simultaneously to avoid distraction by intervening events (unless specifically testing sequential appearance).
  c. Keep effort cost for obtaining a given option constant (unless specifically testing cost).
3. Define the reward and choice variables to be measured.
  a. Infer subjective reward value from observable choice (‘subjective’ refers to the value an individual agent attributes to a reward, not to subjective experience):
    i. The reward chosen more frequently than its alternative(s) has more subjective value than its alternative(s).
    ii. Increase of the reward amount of one option while keeping its alternative(s) constant results in monotonically increasing frequency of choosing that option.
    iii. Model choice frequency psychophysically as choice probability. Typical choice functions are sigmoid, including softmax, Weibull and Gaussian functions.
    iv. Estimate the choice indifference point from the choice function (e.g., with a binary option set, each option is chosen with P = 0.5).
    v. Consider that all options have the same subjective value (utility) at the choice indifference point.
  b. Consider that observable choice reveals also reward preference.
    i. In an experimental setting with repeated trials, define preference for a given option as the probability of choosing that option over all other options in the same option set (stochastic choice theory.^9^^,^^10^^,^^11^
    ii. Consider that the preference of one option to all other options in the same option set, as inferred from choice probability, indicates higher subjective value (utility) of the preferred option compared to all other options in the same option set.^8^
    iii. Consider that preference corresponds to subjective value (utility): higher preference indicates higher subjective value.

**CRITICAL:** Value comparison at choice indifference (P = 0.5 each binary option), rather than at other choice probabilities, is independent from the slope of the choice function.

4. Consider concepts for choice of multi-component options (Revealed Preference Theory^12^) that are suitable for controlled experimental tests in humans and monkeys.
  a. Typical choice options have multiple components, like taste and amount, that vary independently.
  b. The subjective value (utility) of a two-component option depends on the amount of both of its rewards.
  c. Two options with different amounts of their two reward components can have equal value and be equally preferred when reward amounts provide compensation: the higher amount of one component in one option can compensate for the lower amount of the other component of that same option.

### Institutional permissions: Human participants

**Timing: 3 months**

***Note:*** Our study was approved by the Cambridgeshire Research Ethics Committee (REC) of the Health Research Authority (HRA) of the UK National Health System (NHS) (Human Neuroimaging of Reward Processing, 04/Q0108/190).

5. Obtain approval for work on human participants from the local ethics committee.

***Note:*** Unless planned otherwise, ethics requests should exclude clinical investigations whose approval process is more demanding and can take more time.

6. Recruit participants.

***Note:*** In a university setting, posters around campus provide the easiest access to participants. Respect conditions of ethics approval for participant payment.

7. Select participants.

***Note:*** Signals from functional magnetic resonance neuroimaging (fMRI) outside the visual system are usually too weak to be statistically significant in individual participants and thus require averaging across multiple participants (usually at least N = 20; obtain more precise numbers from power analysis). Therefore, participants should have similar characteristics, including sex, gender, age, risk attitude, and stated preferences. If participants of both sexes or genders are required for equality reasons, additional analyses (e.g., regressors) are required for group comparisons.

8. Consider participants’ appreciation of the planned reward liquids, as well as their tolerance of the physical delivery system. Select acceptable rewards and delivery system in preliminary tests.
9. Exclude participants with health or behavioral issues, including those with neurological, psychiatric and metabolic disorders (unless these disorders form a part of the study.

### Institutional permissions: Monkeys

**Timing: 2–12 months**

***Note:*** Our study was approved and supervised by the UK Home Office; the local UK Home Office Inspector; the UK Animals in Science Committee; the UK National Centre for Replacement, Refinement and Reduction of Animal Experiments (NC3Rs); the University of Cambridge Animal Welfare and Ethical Review Body (AWERB); and the Cambridge University’s Biomedical Service (UBS) Certificate Holder, UBS Welfare Officer, Named Veterinary Surgeon (NVS) and Named Animal Care and Welfare Officer (NACWO).

10. Ethical and practical approval:
  a. Explore the feasibility of the research plan, and its possible adaptation, for acceptance by local and national ethical review committees.
  b. Explore feasibility and support at the institution. Consider presence of trained and certified regulatory personnel.
  c. Apply for an institutional and/or government animal experimental license, which may require several months of writing, commenting, discussing and approving by local and national committees.
11. Housing, care and welfare:
  a. Assure housing in enriched cage environment and in small groups that allow social interaction (e.g., grooming) but are not too big for provoking fights. Group sizes of 2–4 animals often turn out well.
  b. use of liquid reward as the most straightforward method for delivering precise reward quantities. In most cases, the method requires fluid control, which presents no problem with decent management but can be an issue for ethical committees and personnel unfamiliar with its practicalities. As an alternative, food can be liquidized and delivered via a peristaltic pump; the method is hampered by less precise quantification but requires only food and not fluid control. After several attempts, we renounced liquidized food and went back to liquid rewards.

**CRITICAL:** Monkeys are sentient and precious animals, research on them is important, and neurophysiology on behaving monkeys is demanding. Therefore, respect of animal welfare is not only ethically required but also assures animal cooperation and thus benefits the research. This pays off for the experiment: it is amazing how well comfortable and relaxed monkeys perform demanding tasks for several hours on a regular daily basis.

12. Purchase health-tested monkeys from well-managed breeding centers.
  a. Visit the center personally to select every animal for friendly interaction with humans and conspecifics.
  b. Consider using animals that are pre-trained with stimuli (e.g., ‘clicker training’), which will save time but may be problematic if use of own stimuli is planned (extinction does not erase memory completely).

## Key resources table

**Table:** 

REAGENT or RESOURCE	SOURCE	IDENTIFIER
**Chemicals, peptides, and recombinant proteins**		

^a^Whole milk (3.5% fat)	Standard supermarket	N/A
^a^Double cream (48% fat)	Standard supermarket	N/A
^a^Sugar (granulated)	Standard supermarket	N/A
^a^Skimmed milk (0.3% fat)	Standard supermarket	N/A
^b^Blackcurrant juice (0–0.8 mL / trial)	Standard supermarket	Ribena
^b^Grape juice (0–0.8 mL / trial)	Standard supermarket	N/A
^b^Water (0–0.8 mL / trial)	Standard supermarket	N/A
^b^Apple juice (0–0.8 mL / trial)	Standard supermarket	N/A
^b^Mango juice (0–0.8 mL / trial)	Standard supermarket	N/A
^b^Monosodium glutamate (MSG; 20–50 mM)	Sigma-Aldrich	G1626
^b^Inosine monophosphate (IMP; 2–5 mM)	Sigma-Aldrich	57510
Electrode Gold Plating solution (#1; 10 mL)	NeuraLynx, Bozeman, MT, USA	N/A
Electrode Platinum Black Plating solution (#2; 25 mL)	NeuraLynx, Bozeman, MT, USA	N/A

**Experimental models: Organisms/strains**		

Human participants (50% male, 50% female; 19–36 years old)	University and local adverts	N/A
Macaca mulatta (male, initial age 3–4 years)	Centre for Macaques (CFM), UK	N/A

**Software and algorithms**		

Matlab w/Data Acquisition Toolbox (1 / computer)	MathWorks Inc	N/A
Psychtoolbox	http://psychtoolbox.org/	Version 3
SPM 12 data analysis package (1 / analysis computer)	Wellcome Trust Center for Neuroimaging, London	N/A
Offline Neurophysiological Spike Sorter (1 / researcher)	Plexon	Versions 1.0–4.0

**Other**		

Reward delivery system for humans with components:	Cambridge University workshop	Custom-made
Piston pump for human reward delivery	New Era Pump Systems www.syringepump.com	NE-500
50 mL syringe for piston pump for humans	Standard medical supplier	N/A
Mouthpiece with pipette tips for liquid reward delivery	Cambridge University workshop	Custom-made
Single-use pipette tips for liquid reward delivery	Standard medical supplier	N/A
Silicon tubes to connect mouthpiece to piston pump	Standard medical supplier	N/A
Dell computer for human data acquisition	Dell Inc	N/A
Data acquisition card for Dell computer	National Instruments Inc	NI-USB-6009
Human fMRI scanner	Siemens	3T Magnetom Skyra
Primate chair (1 / monkey)	Crist Instruments Inc, Hagerstown, MD, USA	Customized TV chair model
Touch-sensitive computer monitor for stimulus presentation and registering the monkey’s choice	EloTouch	1522L 15″
Computer monitor stand with fixation to primate chair	Cambridge University workshop	Custom-made
Joystick hand manipulandum for monkey	Cambridge University workshop	Custom-made
Touch-sensitive holding key for monkey	Fribourg University workshop	Custom-made
Electromagnetic solenoid valve for liquid reward delivery	Standard commercial supplier	N/A
Monkey recording chamber (40 × 40 mm top area; 1 / monkey)	Gray Matter Research, Bozeman, MT, USA	N/A
Tungsten neurophysiological electrodes (length 125 mm; shank 125 μm; angle 60 deg; impedance < 1 MΩ)	Alpha Omega	366-125610-00
Metal guide cannula made from spinal needle (0.6–0.8 outer diameter)	Standard commercial supplier	N/A
Single-electrode drive	Narishige, Japan	MO-97
Single-microelectrode preamplifier	Fribourg University electronics workshop	Custom-made
Differential neuronal impulse amplifier with low-pass filter (1 / electrode)	Bak Electronics Inc Umatilla FL, USA	DCDA-1
Time – amplitude window discriminator (1 / electrode)	Bak Electronics Inc Umatilla, FL, USA	DDIS-1
Oscilloscope for monitoring neuronal discharges	Tektronix	TDS 3014B
Multi-electrode drive (chamber mount; 2-5 electrodes)	NAN Instruments	N/A
Multi-electrode preamplifier (1 / electrode)	NAN Instruments	N/A
Dell computers: N = 1 for monkey behavioral control, N = 1 for monkey neurophysiological data acquisition	Dell Inc	N/A
Data acquisition card ( 1 / computer)	National Instruments Inc	NI-USB-6009

Note: ^a^Distinct reward liquids for humans (2 liquids/milkshake); ^b^Distinct reward liquids for monkeys (1 liquiid/reward).

## Materials and equipment

### Distinct reward liquids for humans (2 liquids/milkshake)

• Low sugar - high fat milkshake (0–10 mL / trial) containing a mixture of whole milk (25%) and double cream (75%).
• High sugar - low fat milkshake (0–10 mL / trial) containing a mixture of sugar (10%) and skimmed milk (0.3% fat).

### Human laboratory

**Timing: 2–6 months**

• Find a quiet room without interference by external noise and other individuals (i.e., individual testing, no presence of researcher).
• Set up a desk or table with computer and keyboard, and a comfortable chair.
• Set up a reward delivery system:
  ○ Select a simple system that can deliver well-quantifiable liquid reward directly into the participant’s mouth via a custom-made mouthpiece. Try single-use pipette tips onto which the participant bites.
  ○ Connect the mouthpiece to silicone tubes approved for delivery of reward liquids to humans. For the two distinct rewards, attach two tubes to two respective 50 mL syringes, each driven by a separate piston pump. Each pump delivers a computer-programmable amount of one liquid with milliliter precision via a National Instruments card and the Matlab Data Acquisition Toolbox.
  ○ Reward piloting: To initially obtain quantitative indications about participants’ sensitivity to reward liquids, measure simple binary choice of single-component options using a wide range of different liquids and amounts. Select liquids for which participants show choice probabilities fully between P = 0 and P = 1. This procedure can take several weeks or months of testing during every working day of the week.

### Monkey laboratory

**Timing: 2–12 months**

• Set up a monkey laboratory for controlled behavioral tests and neurophysiological recordings. It should contain:
  ○ A touch-sensitive computer monitor for stimulus presentation and registering the animal’s choices, fixed rigidly to a solid stand.
  ○ A hand or arm manipulandum, such as a joystick and/or a touch-sensitive key, fixed rigidly to the same stand.
  ○ A primate chair in which the animal is comfortably seated. Each animal should have its own, carefully adjusted primate chair for the whole duration of the experiment.
  ○ A platform on wheels for positioning and moving the animal inside the chair from the animal house into the laboratory, and for daily testing in the chair.
  ○ A rigid mechanical connection between the monitor stand and the primate chair.
  ○ A screen or an enclosure that separates the animal together with the monitor stand from the experimenter and the rest of the laboratory. An enclosure should provide sound attenuation.
  ○ An electromagnetic solenoid valve for well-quantifiable delivery of liquid reward.
  ○ Mechanical and electronic equipment for delivering rewards, controlling and measuring the animal’s behavior, and for neuronal recordings.

***Note:*** Before modifying an existing laboratory or setting up a new laboratory, obtain latest hands-on knowledge, including names of purveyors of equipment and material, by visiting a colleague’s laboratory.

***Note:*** Monkeys are the closest species to humans in which the activity of individual neurons can be studied on a routine basis, in a freely chosen brain structure, in an ethically acceptable way, and unrelated to pathological states. Such monkey investigations help to understand the human condition and the brain systems impaired in human diseases. Macaque monkeys can perform sufficient trial numbers in specifically designed behavioral tasks necessary for statistics, well beyond observation of on-going behavior. The tasks allow identification of neural reward signals by finely controlling for somatosensory and visual stimulation and eye, limb and trunk movements. Suitable species are the rhesus monkey (Macaca mulatta) who usually shows solid task performance but also a strong sense of hierarchy that can lead to group fights with serious injuries, and the long tailed Java monkey (Macaca fascicularis) who may show less stable task performance but also less fighting. The common marmoset (Callithrix jacchus), who generates less antivivisectionist interest and requires less space, is suitable for some behavioral tasks and allows neurophysiological tests if sensory and movement control can be assured. The well-controllable behavior of monkeys contrasts advantageously with the behavior of rodents who often show inadvertent task-unrelated movements and brain-wide sensory and motor signals.^13^^,^^14^

## Step-by-step method details

### Common behavioral methods for humans and monkeys

**Timing: 2–12 months**

Use the economic concepts for designing behavioral tasks suitable for assessing the participant’s choices and estimate their subjective reward value and preference. The task should be appropriate for identifying time-specific, discriminant and well-interpretable neural signals in discrete, repeated trials with well-defined onset and end. Each trial contains a limited number of temporally well-separated events, such as reward-predicting stimuli, movement and reward. Adequate temporal separation of task events allows us to analyze neural signals without overlap by other task events, thus reducing intercorrelations. The statistical analysis of neural responses requires repeated trials, which allows us to apply the concepts of stochastic choice theory mentioned above (^9^^,^^10^^,^^11^). Only major steps of the design are presented here; for details see our earlier study.^3^

**Figure 1:**
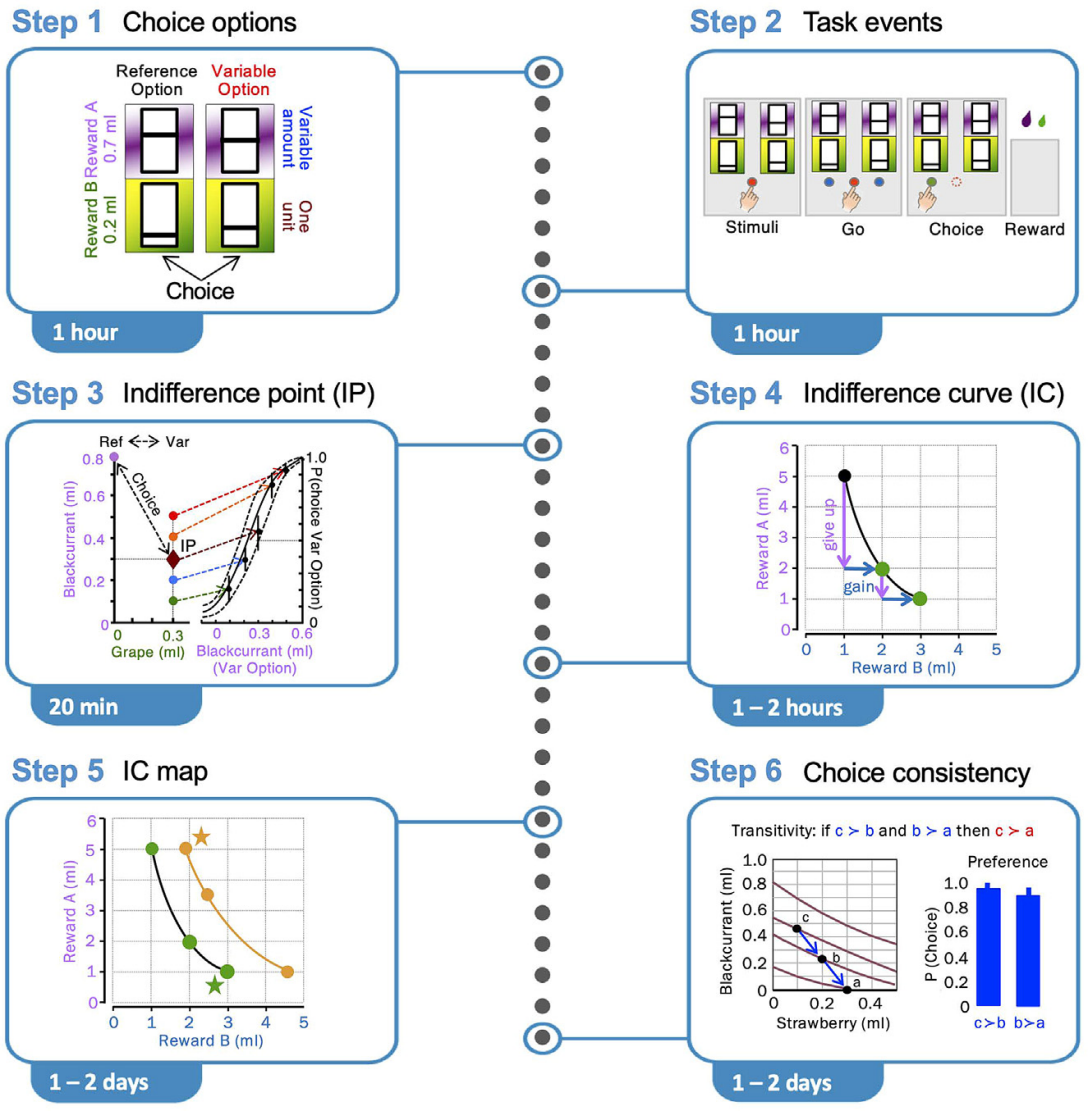
Step-by-step schematics of concept-driven behavioral methods (Step 1) Define the choice options. Quantitative stimuli predict two options, each of which contains the same two reward liquids indicated by colors (blue, green). Their independently set amounts are indicated by vertical bar position within each rectangle (higher is more). (Step 2) Define the sequential task events (example for monkeys). Key touch triggers appearance of the quantitative stimuli on a computer touch monitor. Subsequent touch of a central spot (red) elicits appearance of two lateral spots (blue). Touching one of the lateral spots results in delivery of the two rewards of the chosen option. (Step 3) Estimate subjective reward value at choice indifference point (IP). Left: example choice between preset Reference Option (Ref: blackcurrant juice 0.8 mL, grape juice 0 mL) and Variable Option (Var: blackcurrant juice 0.1–0.5 mL, grape juice 0.3 mL). Right: psychophysics: choice probability for Variable Option increases with blackcurrant juice being given up (Weibull fit ± 95% confidence interval). At choice indifference (P = 0.5 each option), at which both options have same subjective value, the animal gives up 0.5 mL of blackcurrant juice to gain 0.3 mL of grape juice. (Step 4) Estimation of choice indifference curve (IC) from multiple choice indifference points (IP). At each IP, the animal gives up some amount of Reward A (e.g., blackcurrant juice) to gain some amount of Reward B (e.g., grape juice) without change of value. (Step 5) IC map composed of two ICs. All options on a same-colored IC have same subjective value. All options on a higher IC (farther away from origin) have higher value compared to all options on a lower IC, despite different reward composition. The value difference between ICs holds even when one component of a higher-IC option is smaller than in a lower-IC option (partial physical non-dominance; stars). (Step 6) Transitivity tests on ICs. Brown curves show best-IC-itting quadratic polynomials. Preferences of options were inferred from IC order as c ≻ b ≻ a. Graphs in all steps are adapted from our earlier study.^3^

***Note:*** The presentation of symmetric choice options that each have two rewards reduces visual confounds and allows more focused data analysis, as compared to more ‘natural’ options with heterogeneous components.

1. Define the choice options (Figure 1 step 1).
  a. Use two options (binary choice), a pre-settable Reference Option and a Variable Option with one psychophysically adjustable reward.
  b. Use two components in both options.
  c. Select two specific reward liquids for each option.
  d. Use the same two reward liquids in each option.
  e. Set each reward in each option to a specific amount.
  f. Consider that the subjective value (utility) of a two-component option depends on the amount of both of its rewards.

2. Define the task events (Figure 1 step 2).
  a. Trial start.
    i. Humans: the participant is presented with a small cross at the center of the computer monitor. The cross generates attention and thus focuses the eyes on the center, usually without requiring specific ocular control.
    ii. Monkeys: the animal contacts a touch-sensitive key to start a trial without being prompted by a specific stimulus on the computer monitor.
  b. Simultaneous appearance of two visual stimuli on the computer monitor, each stimulus indicating a two-component choice option.
    i. Each stimulus alternates pseudorandomly between left and right stimulus positions to avoid side bias (and control for spatial neural coding).
    ii. Each stimulus contains two vertical rectangles.
    iii. The bar in each vertical rectangle indicates a specific reward amount (higher is more).
  c. Choice between two options.
    i. Humans: the participant presses one of two specific keys on a computer key board.
    ii. Monkeys: the animal releases the touch-sensitive key and contacts one of two specifically marked areas on the computer touch monitor with its hand to choose the option indicated by the respective stimulus.
  d. Rewards: the two rewards of the chosen option are paid out at trial end according to the specific schedules for humans and monkeys.
  e. The next trail starts after an inter-trial interval of 1.6 ± 0.25 s.

***Note:*** The complexity of the visual stimuli must not exceed the visual capacity of humans or monkeys.

***Note:*** The repeated delivery of reward induces a Pavlovian association between each visual stimulus component and the type and amount of each reward liquid. Thus, each stimulus component becomes a predictor of the specific liquid and amount.

3. Estimate a choice indifference point (IP) between two two-component choice options (Figure 1 step 3). At the IP, each option is chosen with P = 0.5 (binary option set).
  a. Define one two-component option as Reference Option and set both of its rewards to specific test amounts (Figure 1 step 3, Ref at top left).
  b. Define the other two-component option as Variable Option (Var).
    i. Set one of its rewards to a specific test amount.
    ii. Pseudorandomly vary its other reward across the full range of testable reward amounts, using several fixed amounts (Figure 1 step 3, left).
  c. Psychophysically estimate a choice indifference point (IP) at which the Reference Option and the Variable Option are equally preferred (Figure 1 step 3, right). Proceed as follows:
    i. Repeatedly measure the choice frequency for each amount of the variable reward of the Variable Option, pseudorandomly alternating between the amounts.
    ii. Fit a sigmoid choice function (softmax, Weibull or Gaussian) to the choice frequency.
    iii. Estimate the IP at choice P = 0.5 of each option.

**CRITICAL:** The amounts of both reward components of the Reference Option define its subjective value. Any IP between that Reference Option and any other option (Variable Option) has by definition the same subjective value, even if the reward amounts of the two components of that other option differ from the reward amounts of the Reference Option. Thus, even options with oppositely varying reward amounts can have the same subjective value. This is the way how single-dimensional subjective values (and neuronal signals) emerge from multi-component choice options.

4. Estimate a choice indifference curve (IC) from multiple IPs (Figure 1 step 4).
  a. Keep the same Reference Option but set the Variable Option to a different test amount than before.
  b. Psychophysically establish an IP between the new Variable Option and the original Reference Option.
  c. The new IP has by definition the same subjective value as the Reference Option, and by transitivity the same subjective value as the initial Variable Option.
  d. Pseudorandomly select a new Reference Option from the existing Variable Options and use a new Variable Option to establish a new IP, thus avoiding gradually increasing measurement inaccuracies and systematic deviations due to direction of testing.^15^ Repeat this step a few times for increasing accuracy of the subsequently estimated IC.
  e. Hyperbolically fit an IC to all IPs for the same initial Reference Option. Plot the 95% confidence interval on each side of the IC to indicate the fitting error.
  f. As the Reference Option defines the subjective value of all equally preferred two-component options, all options on the same IC have the same subjective value and are equally preferred to each other despite their different reward amount composition.
  g. The equal preference can be thought of as giving up some amount of one reward for gaining some amount of the other reward without loss of subjective value (Figure 1 step 4, colored arrows).

***Note:*** The coefficients of the estimated IC hyperbola provide the slope and the curvature of the graphic IC. A more simple measure of IC curvature is provided by the maximal vertical distance between the curve and a line connecting the curve intercepts with the y-axis (x = 0) and the x-axis (y = 0); the distance is expressed in ml of the y-axis liquid.

5. Estimate an IC map (Figure 1 step 5).
  a. Set different Reference Options to estimate several different ICs.
  b. All options on a higher IC (farther from origin) are preferred to all options on a lower IC.
  c. Across ICs, an option with one smaller reward component than its alternative can be preferred to an option with a larger reward component if the first option’s other reward component is much larger (partial physical non-dominance) (Figure 1 step 5, stars).

**CRITICAL:** With two-component options, a smaller reward component of preferred option compared to same reward component of alternative option indicates that the subjective value of two-component options results from integrating both components, rather than simply following the reward amounts of all components. This partial physical non-dominance further demonstrates how single-dimensional subjective values (and neuronal signals) emerge from multi-component choice options.

6. Test choice consistency via transitivity using not previously estimated IPs (Figure 1 step 6).
  a. Test choice between two options that are randomly set onto a high IC vs. a middle IC. The high-IC option should be preferred to the middle-IC option (a ≻ b; '≻' indicates preference).
  b. Test choice between that middle-IC option and a low-IC option at a position that is not an IP. The middle-IC option should be preferred to the low-IC option (b ≻ c).
  c. Assess transitivity by testing choice between the high-IC option and the low-IC option. To comply with transitivity, the high-IC option should be preferred to the low-IC option (a ≻ c).
  d. Strong stochastic transitivity^9^ is evidenced by higher preference (probability of choice) for (a ≻ c) compared to both preferences (a ≻ b) and (b ≻ c).
7. For further validity testing, use leave-one-out statistics to assess the contribution of individual IPs to a single fitted IC. Compare the difference between the original IC and four new ICs estimated with one of the original IPs left out, using four criteria.
  a. Lack of overlap between [the new IC fitted without the left-out option] and [another IC].
  b. Lack of overlap between [the new IC fitted without the left-out option] and [the 95% confidence interval of another IC].
  c. Overlap between [the new IC fitted without the left-out option] and [the 95% confidence interval of the original IC].
  d. Insignificant deviation between [the new IC fitted without the left-out option] and [the left-out option].
8. For further validity testing, use out-of-sample tests to confirm a basic assumption of the IC scheme: options on higher ICs have higher subjective value than, and are preferred to, options on lower ICs.
  a. Set each component of one option to a randomly selected position (graphically an x-y point) on a higher IC (farther from origin). The position should not be a previously estimated IP.
  b. Position the other option of the same option set onto a randomly selected non-IP x-y coordinate on a lower IC.
  c. Test repeated choice between the two options to measure choice probability as indicator of the monkey’s preference.
  d. Repeat this procedure a few times with different IC positions. If the monkey fails to prefer the high-IC option to the low-IC option, the IC map is unacceptable and should be re-established from scratch.

***Note:*** Reasons for inverse preferences could be insufficient training and suboptimal testing.

9. Assess the validity of IPs and ICs using a homothetic model.
  a. Estimate a homothetic model from the hyperbolic or quadratic model that fits best all IPs on all ICs of a given two-component option.
  b. Out-of-points prediction.
    i. Select a test IP on an IC of the homothetic model; the IP should not have been used for constructing the homothetic model.
    ii. Psychophysically estimate a new IP against a Reference Option positioned at the y-intercept (x = 0 mL) of the tested IC of the homothetic model.
    iii. Using the same x-coordinate, compare the y-coordinates between the newly estimated IP and the initially selected test IP.
  c. Out-of-curves prediction.
    i. Remove all IPs belonging to one IC from the homothetic fitting procedure and construct a reduced homothetic model from the IPs of the remaining ICs.
    ii. Use the coefficients of the reduced homothetic model to construct a new IC that corresponds best to the left-out IC.
    iii. Select a test IP on that new IC.
    iv. As with the out-of-points prediction, psychophysically estimate a new IP against a Reference Option positioned at the y-intercept (x = 0 mL) of the tested IC of the original homothetic model.
    v. Using the same x-coordinate, compare the y-coordinates between the newly estimated IP and the selected test IP on the new IC.
  d. For both out-of-points and out-of-curves predictions, use the difference in y-coordinates between the newly estimated IP and the selected test IPs as a metric of validity (ml along the y-axis). Smaller differences indicate better contribution of the IP to the IC, and thus higher IC validity for representing subjective value and choice preference.

***Note:*** In sum, the IC map shows the following properties: all options on higher ICs have higher value than, and are preferred to, all options on lower ICs, even when one component of the preferred option has a smaller amount than the alternative option (partial physical non-dominance; Figure 1 step 5, stars). Thus, all options on the red IC are preferred to all options on the green IC, and all options on the green IC are preferred to all options on the blue IC. When transitivity is satisfied, all options on the red IC are preferred to all options on the blue IC.

10. Test the specificity of trial-by-trial choices using a multiple logistic regression: correlate the choices with the amounts of the option components rather than with other factors that are not of primary interest for understanding choices between two-component options, such as reaction time, position of visual stimulus indicating the option, and previous choice.

### Specific behavioral methods for human participants

**Timing: 1–2 months**

Specify the human choice task and adapt it to the requirements for subsequent fMRI neuroimaging. For details see our earlier study.^2^

11. Define reward liquids: each option consists of a mixture of two milkshakes with specific amounts of the same two ingredients, sugar and fat.
  a. milkshake 1: low-sugar high-fat (no sugar, 25% whole milk and 75% double cream).
  b. milkshake 2: high-sugar low-fat (10% sugar, skimmed milk).
12. Collect choice data.
  a. Task training: run several sessions of 10–30 min until stable performance with consistent preferences for two-component options is obtained.
  b. If necessary to prevent rapid satiation, consider paying out reward only on every fifth trial on average (20%), or at a similar frequency, the payout trial being selected from a Poisson distribution with a mean of five (trials). However, intermittent reward delivery likely results in confounds of attention, expectation and risk to be included in the data analysis.
  c. Estimate 4 IPs for each of 3 Reference Options to obtain 3 ICs for the same two liquid components. As each IC requires 7 test amounts and 6 repetitions, the total procedure requires 504 trials in each of > 20 participants.
  d. Including an inter-trial interval of 0.5 s, the 20% rewarded trials last a mean of 11.0 s, the 80% unrewarded trials last a mean of 5.5 s. Thus, the total of 504 trials requires 55 min.
13. Validate the value order of the choice options independently of the estimation mechanism with a Becker-DeGroot-Marschak (BDM) auction-like bidding mechanism.^16^
  a. The participant receives in every trial a new ‘endowment’ of a constant amount of water.
  b. The computer sets pseudorandomly a bid that is not displayed to the participant.
  c. The participant bids for a given two-component option against the computer bid.
  d. If the participant’s bid is higher than or equal to the computer bid, the participant receives both component liquids of the option (‘win’) and ‘pays’ an amount from the water endowment that is equal to the computer bid (second price auction).
  e. If the participant’s bid is lower than the computer bid, the participant loses the auction, pays nothing by receiving the full water endowment, and does not receive the reward s/he bid for.
  f. To start the BDM, present the vertical composite visual stimulus for one two-component option on a computer monitor to the left of a scale of 0–20 UK pence.
  g. To place a BDM bid, the participant moves a cursor horizontally on the monitor, using the left and right keyboard arrows.
  h. Collect BDM bids in each of > 20 participants for 15 options with 12 repetitions, which requires 180 trials in one session lasting 55 min.
  i. Analyze BDM bids according to the IC scheme (Figure 1 step 5).
    i. Test monotonicity across ICs with linear regression.
    ii. Test monotonicity across ICs without requiring linearity with Spearman rank correlation.
    iii. Test significance across ICs and insignificance along ICs with 2-factor Anova (factors 1 and 2, respectively). Use insignificance of the along-IC factor to indicate insignificantly different BDM bids for options positioned on same IC, suggesting similar value of all options represented by a single IC.
    iv. Compare the BDM bids with ICs by hyperbolic fitting of BDM isolines to similar BDM bids and plot the BDM isolines on top of the ICs. Isolines that match the ICs should stay within the 95% confidence intervals of ICs.
14. Use binary machine learning decoders to determine the accuracy of ICs representing the value of choice options.
  a. Test whether a binary support vector machine (SVM) decoder accurately assigns an option to its original IC as opposed to a neighboring IC.
  b. Test whether two-dimensional linear discriminant analysis (LDA) assigns a given test option to one of the different ICs (first discriminant) without assigning it to any particular position on a given IC (second discriminant).
  c. Test whether binary SVM and two-dimensional LDA assign BDM bids correctly to ICs.

### Neuroimaging methods for human participants

**Timing: 2–4 months**

Use the behavioral task, validated by demonstrating meaningful choices, for investigating neural reward signals for multi-component choice options in specific human brain regions using fMRI neuroimaging. For details see our earlier study.^1^

15. Prepare fMRI scanning.
  a. Fix dates for fMRI scanning with local imaging center, typically for a 3-Tesla scanner.
  b. Confirm usefulness of a previously validated scan sequence^17^ or else establish a new scan sequence.
  c. In the scanner, tilt the scan acquisition plane by 30° to reduce signal dropout in medial-temporal and inferior frontal regions.
  d. Once the fMRI technology is assured, set scan dates with all participants from the behavioral tests.
    i. Ideally, scan soon after participants show consistent behavioral performance.
    ii. Recruit and task-train additional participants if necessary.

***Note:*** Statistical analysis of the small fMRI signals requires data averaging over multiple trials and multiple participants anchored to discrete task events (stimuli, action, rewards). Therefore, the collection of sufficient fMRI data for each participant may require two sessions of 30–45 min each on different days.

***Note:*** Data averaging across multiple participants involves a compromise between general economic principles and reliable data analysis: preferences and utility are subjective and thus cannot be easily compared between participants. To reduce confounds, the participants should be matched for sex, gender, age and risk attitude.

**CRITICAL:** All participants must be tested in the same scanner to allow data averaging across participants.

16. Adapt behavioral task to scanning conditions.
  a. To assure the participants’ cooperation in the noisy scanner, split the behavioral test session of 55 min into two shorter scanning sessions on two consecutive days.
  b. Habituate each participant to task testing in a horizontal position, ideally using a mock scanner with comparable scanning noise.
  c. Re-test the task in each participant and adjust the amount of both option components until all participants show similar ICs to facilitate data averaging across participants.
17. Record fMRI neuroimaging data.
  a. To eliminate confounds from the unchosen option, collect event-related fMRI neuroimaging data only during no-choice trials. Only one two-component option is shown in these trials.
  b. Scan in individual sessions of 30–40 min duration / participant.
  c. Perform second scan on another day soon after the first scanning day.
18. Use the SPM 12 data analysis package for preprocessing.
  a. Realign the data to correct for motion.
  b. Normalize the data to standard Montreal Neurologic Institute (MNI) coordinates.
  c. Smooth data using a Gaussian kernel with the full width at half maximum (FWHM) of 6 mm.
19. Use SPM 12 to set up and run an initial General Linear Model (GLM) to identify blood-oxygen-level-dependent (BOLD) signals in brain regions of interest (ROI) whose stimulus-induced activations follow the two-dimensional IC scheme (Figure 1 step 5).
  a. Variation across ICs (high > low).
  b. Similarity along same ICs.
20. Alternatively, apply small volume correction based on published data from a large number of studies on human fMRI neuroimaging responses to reward-predicting stimuli (> 50) to define ROIs measuring 6–10 mm in radius.

***Note:*** The most reliable fMRI ROIs with responses to reward-predicting stimuli are found in ventral striatum, midbrain, and orbitofrontal and ventromedial prefrontal cortex.

21. Use Spearman rank correlation to test for monotonic response change across the three ICs within each ROI.

***Note:*** The initial option-predicting stimulus contains simultaneous and full information about both components of the option, whereas the final individual two outcomes occurring with 0.5 s interval provide only partial information about the option (for sequential task events, see Figure 1 step 2). Thus, to capture the integrated value of the options, focus the data analysis on responses to the initial stimulus rather than on the ultimate delivery of the individual reward components

22. Run another GLM using SPM 12 to further assess meaningful responses.
  a. Identify brain regions with stronger response even when one of the components in the preferred option (on a higher IC) is smaller than in the alternative option (on a lower IC) (partial physical non-dominance) (Figure 1 step 5, stars).
  b. Subsequently directly compare the fMRI neuroimaging responses between the two options on different ICs.
23. Analyze BDM bids with a GLM using SPM 12. Identify brain regions whose fMRI neuroimaging responses to stimuli for individual options correlates with BDM bids during the bidding phase.

### Specific behavioral methods for monkeys

**Timing: 3–9 months**

As with the human part of the study, use the economic concepts to design a choice task in monkeys that is suitable for assessing subjective value and preference for multi-component choice options. The tasks should be appropriate for subsequently investigating the underlying neuronal signals. For details see our earlier study.^3^

24. Define reward liquids.
  a. Each option contains two liquids, such as blackcurrant juice, grape juice, water, apple juice or mango juice.
  b. To enlarge the range of testable rewards, add taste enhancers, such as monosodium glutamate (MSG; 20–50 mM) or inosine monophosphate (IMP; 2–5 mM).
  c. In every correctly performed trial, always pay out the two rewards of the chosen option (reward probability P = 1.0; ‘safe rewards’) and in a constant sequence, separated by 0.5 s.
25. Habituate each animal to controlled testing in a primate chair.
  a. Separate the animal from the other animals in the home cage for each daily procedure.
  b. Position and secure the chair in front of the door of the animal’s home cage.
  c. Place a tunnel in front of the cage to facilitate passage of the animal into the chair (a cover on top of the chair prevents escape into the room).
  d. Place attractive food items into the tunnel and chair, each day gradually farther away from the home cage, to habituate the animal to entering the tunnel and then the chair.
  e. When the animal enters the chair completely via the tunnel, each day touch the neckplate of the chair to habituate the animal to such manipulations. The animal should not withdraw its head into the interior of the chair.
  f. Close the neckplate of the chair and immediately administer attractive foods and/or liquids as rewards.
  g. Check the chair for comfortable fit and adjust over several days to achieve completely pressure-free fitting that can be sustained over several hours.
  h. After well entering the primate chair, wheel the animal inside the chair, over several days, gradually further towards the laboratory.
  i. Release the animal into its home cage each day, and immediately give additional attractive foods and/or liquids.
  j. Do final adjustments of the primate chair for comfortable fit and pressure-free seating.

**CRITICAL:** Do not advance too rapidly to avoid setbacks.

**Figure 2:**
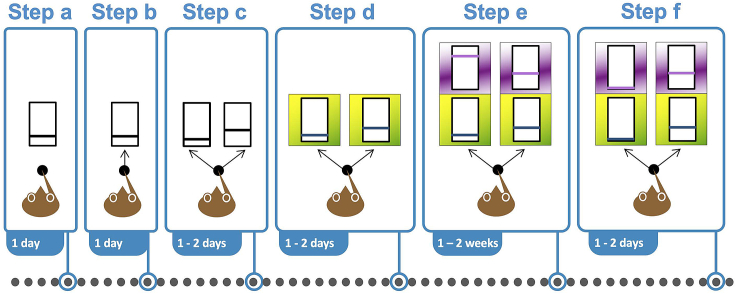
Step-by-step task training for monkeys (Step a) Pavlovian stimulus conditioning. The animal touches a resting key and without further action receives a single reward with a specific amount (height of bar in vertical rectangle; higher is more). (Step b) The animal releases the resting key and touches the stimulus to get the reward. (Step c) The animal chooses between two stimuli predicting different amounts of the same reward liquid. (Step d) Specific colors and textures surround the quantitative stimuli for specific reward liquids. (Step e) Complete task stimuli: each choice option contains the same two reward liquids (indicated by different colors) with independently set amounts. (Step f) No-choice control: both rewards of one option are set to 0. Graphs in all steps are adapted from our earlier study.^4^

***Note:*** Gradually advance over several weeks to include all stimulus and reward settings of the final task. Accept daily variations but keep performance level above 75% correct.

26. Initiate task training by requiring the animal to perform a simple action that forms a component of the ultimate full task, like touching a resting key or touching a specific spot on a computer monitor to receive a drop of liquid reward.
27. Step-by-step task training (Figure 2).
  a. Pavlovian conditioning (Figure 2 step a): a simple quantitative reward stimulus presented on the computer monitor predicts a specific liquid amount according to the vertical bar position of a horizontal bar inside a rectangle (higher is more, a universal metaphor also familiar to monkeys). Deliver a single reward with the indicated amount without requiring an action by the animal.
  b. Operant conditioning (Figure 2 step b): now require the animal to touch the stimulus on the computer monitor to obtain the indicated reward amount. Thus, the operant is the arm movement, and the animal is free to move its eyes.
  c. Choice (Figure 2 step c): present two quantitative stimuli, each predicting a different amount of the same reward. The animal chooses by touching one, and only one, of the two stimuli.
  d. Add to the quantitative stimuli a specific color or background to unambiguously identify each specific reward liquid (Figure 2 step d). Use the same color or background for the same liquid in both options.
  e. Add a second component to each option that is identified by a different color or background (Figure 2 step e).
  f. No-choice task with visual symmetry (Figure 2 step f), for avoiding interference from choice and unchosen option: set the amount of both rewards of one option to zero. Train the animal until it no longer chooses this option.
  g. At every step, check correct understanding of quantitative stimuli leading to meaningful choices.
    i. The animal should choose the larger reward in > 50% of trials (stochastic choice).
    ii. Choice of the smaller option in < 50% of trials indicates incomplete training that should dissipate with continuing task experience (remaining 5%–10% choices of lower amounts would comply with stochastic choice theory).
    iii. When changing the reward amount of an option, the animal should select the better reward on the very first trials.
  h. Train the animal until it reaches about 500–800 correct trials on each test day.

28. Estimate IPs, ICs and IC map.
  a. Estimate an IP with 5 settings of the Variable Option against the same Reference Option, with left-right alternation of stimulus position and 8 trials per position, requiring a total of 5 × 2 × 8 trials = 80 trials / IP.
  b. Estimate an IC with 5 IPs, requiring a total of 5 × 80 trials = 400 trials / IC.
  c. Estimate 2–6 ICs to obtain a full IC map.
29. Estimate IPs, ICs and IC maps for several different reward liquids. Keep one reward the same to establish a common currency in which the subjective value of all other rewards can be scaled.
30. Test relative reward-specific satiety to check how value change affects ICs.

***Note:*** Macaque monkeys perform well when tested by the same researchers or technicians at the same time on every day of the working week. Their cooperation and performance increases gradually over several weeks of stereotyped testing. Therefore, avoid changing personnel and varying experimental hours (‘monkeys hate changes’).

#### Neurophysiological methods for monkeys

**Timing: 6–12 months per animal (1–2 years for a minimum of two animals)**

Use the behavioral task, validated by meaningful choices, for investigating reward signals for multi-component choice options in single neurons of specific brain structures of macaque monkeys. For details see our earlier study.^4^^,^^5^

31. Select brain structures of interest.
  a. Orbitofrontal cortex (OFC): only subsets of OFC neurons carry reward signals. Their distinction from non-reward signals may be easy during on-line monitoring but needs to be confirmed by off-line multi-variate data analysis.
  b. Dopamine neurons: besides their pronounced reward prediction error signal, these neurons carry also non-reward signals related to stimulus saliency and behavioral activation^18^ that need to be distinguished by experimental design, careful inspection (maximal response duration of 0.1–0.8 s) and data analysis.
  c. Other possible brain structures.
    i. Amygdala: besides their fear and aversive processing, amygdala neurons carry substantial reward signals^19^^,^^20^ that need to be distinguished from non-reward signals by varying reward amounts while keeping sensory stimuli and movements constant.
    ii. Striatum: most neurons in the caudate nucleus and accumbens, and about half of the neurons in the putamen, carry reward information, either for reward alone or for reward together with movement.^21^
    iii. Frontal, parietal and temporal cortex and several basal ganglia nuclei process reward information together with sensory or motor information.
32. Adapt the behavioral task to the requirements of neuronal testing.
  a. Number of trials per neuron: statistical neuronal data analysis requires multiple trials.
    i. Student’s t-test and Wilcoxon paired-test require at least 10 trials in each tested condition for reasonable significance (e.g., two different reward amounts).
    ii. Other, non-parametric tests usually require at least 20–30 trials per condition for reasonable significance.
    iii. Standard multiple linear regressions should include a regressor for every possible stimulus and behavioral variable in a single model; the number of trials must exceed the number of regressors.
  b. Trial duration.
    i. Short intra-trial durations of 5–10 s allow efficient data collection.
    ii. Longer inter-trial intervals than intra-trial durations assure good association between rewards and stimuli and movements. However, after extensive experience and satisfactory task performance, inter-trial durations can often be shortened to 4–5 s.
    iii. Thus, cycle time (intra-trial plus inter-trial durations) sums to 10–15 s in each trial, which allows 4–6 trials per minute and requires about 10–20 min for recording 40–120 trials from a given neuron.
  c. Train the animal in the final task until 90%–95% correct performance is achieved.
33. Prepare neurophysiological recordings.
  a. Surgical implantation: implant the following devices under general anesthesia and aseptic conditions.
    i. Head holder: for head fixation to allow eye position monitoring, which is also helpful for keeping the animal’s attention on the task and its stimuli.
    ii. Recording chamber: for holding the microelectrode drive.
  b. Electrodes.
    i. Buy glass-covered tungsten recording microelectrodes that are sufficiently rigid to sustain mechanical stress during insertion into the brain and during movement inside the brain. The glass insulation allows us to use precisely shaped and insulated electrode tips for recording from well-isolated single neurons as opposed to poorly separated multiple neurons.
    ii. Platinize the microelectrode tip^22^ to improve the signal-to-noise ratio and thus the identification of the characteristic extracellular discharge of dopamine neurons and their distinction from discharges of neighboring non-dopamine neurons (see below).
  c. Find the position of OFC by implanting the recording chamber at stereotaxic coordinates from the Paxinos brain atlas for rhesus monkey.^23^
  d. Find position of dopamine neurons (Figure 3).
    i. Implant the recording chamber, oriented parallel to the three stereotaxic planes, at 8–10 mm anterior to the interaural line (AP = 8–10). The chamber position defines a new, head-centered stereotaxic reference.
    ii. After a post-implantation recovery of two to three weeks, anesthetize the animal again and perform lateral and frontal radiography of the head with a metal (radio-opaque) guide cannula inserted vertically into the brain (according to the stereotaxic plane) at an antero-posterior position of the substantia nigra determined in reference to the recording chamber.
    iii. Identify the guide cannula on the radiography and then use bony landmarks to calculate the approximate antero-posterior position of the face area of the somatosensory ventroposteromedial thalamus (VPM) that lies above the lateral substantia nigra.^24^
      ***Note:*** the VPM lies about 17–20 mm posterior to the kink in the sphenoid bone, somewhat depending on brain size (which correlates loosely with non-obese body weight).
    iv. One week after radiography, anesthetize the animal again, bring it into the laboratory and fix it in the sphinx position. Immobilize the head atraumatically above the body axis using the implanted head holder.
    v. Insert a recording microelectrode inside a guide cannula vertically into the brain at the approximate position of the VPM face area based on the radiography.
    vi. Locate the VPM face area by electrophysiological recordings. VPM neurons have very small receptive fields (sometimes only one facial hair), very low stimulation thresholds and often tonic responses during maintained tactile stimulation. They are found over a 2–3 mm vertical extent.^25^
    vii. Repeat the VPM mapping in a horizontal grid of 1 mm to identify approximate VPM boundaries. Repeat and extend the mapping once a week to delineate the extent of the VPM. Usually 4–6 electrode tracks are sufficient.

**Figure 3:**
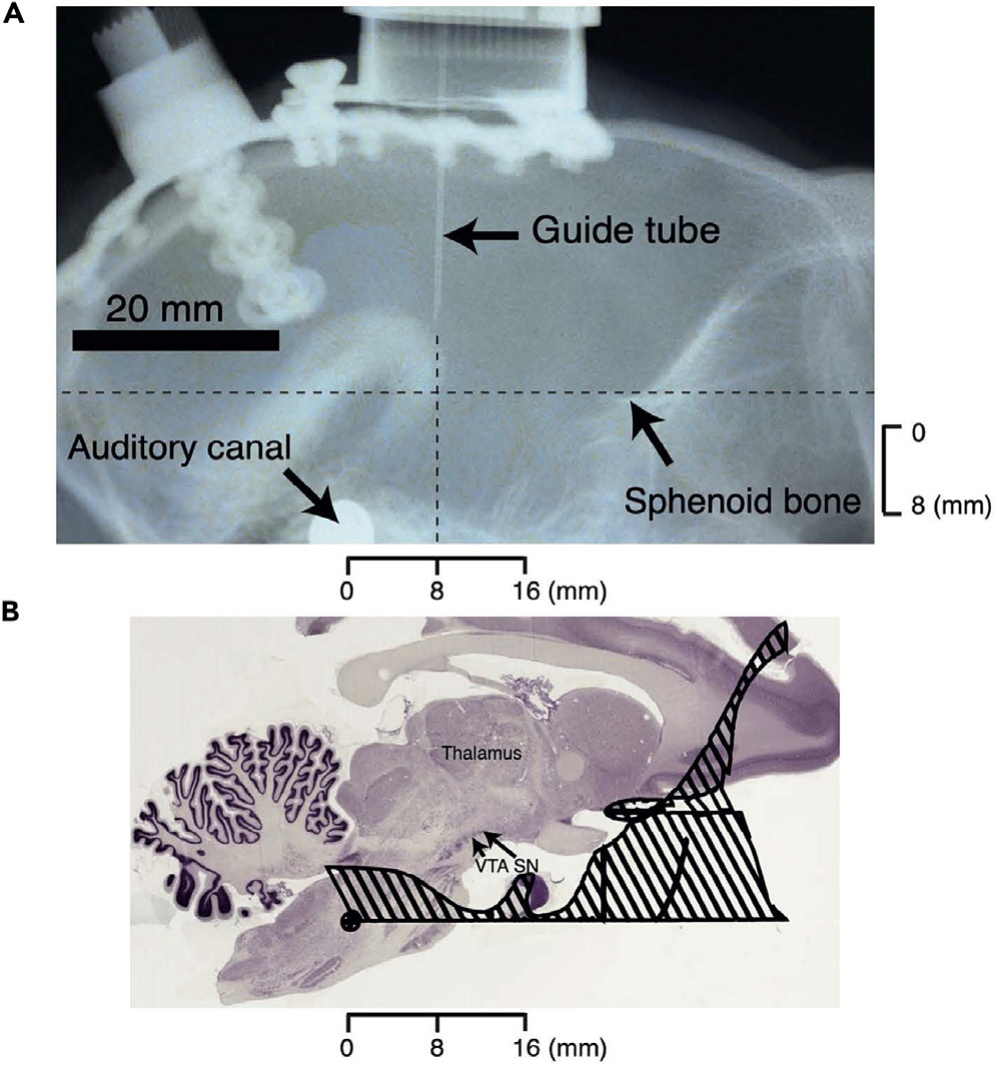
Recording sites of dopamine neurons (A) Lateral radiographic view of a monkey skull with a guide tube directed toward the midbrain. (B) Composite figure of recording area in midbrain. The schematic drawing of the base of the skull was obtained from an earlier study.^28^ Nissl-stained standard sagittal histological section from Macaca mulatta was obtained from www.brainmaps.org (slide 64/295). All figure components are displayed at the scale of the radiography shown in A. Adapted from our earlier study.^24^
34. Collect neuronal data during behavior on most working days of the week.
  a. Bring the animal from the home cage into the laboratory.
  b. Fix the animal’s head and clean the implant to prevent infections.
  c. Insert one or several microelectrodes inside guide tube(s) through the dura into the brain of the awake animal.
  d. Move the microelectrode(s) inside the brain with a micromanipulator to search for, isolate and record from single neurons during task performance. Use a Narishige microdrive for a single electrode and a NAN Drive for multiple electrodes.
  e. Aim for at least 2:1 signal-to-noise ratio of perfectly isolated single neuron action potentials (signal size measured as action potential amplitude), which allows on-line time-window discrimination and digitalization as time events. Multi-unit recordings of 2:1 signal-to-noise ratio require off-line sorting.
  f. Record for at least 20 trials in each of at least 2 trial types. Extend recording durations as long as recording quality remains acceptable (2:1 signal-to-noise ratio).
  g. Repeat searching for, isolating and recording from single neurons until the animal’s daily performance degrades. Recording durations are usually 2–4 h each day, during which the animal performs 500–800 correct trials.
  h. Respect the heterogeneity of neuron types.
    i. Extracellular neurophysiological recordings in OFC can distinguish between different response types but cannot identify their anatomical identity.
    ii. Extracellular recordings from dopamine neurons in substantia nigra pars compacta and ventral tegmental area serve to determine their identity and distinguish against neighboring non-dopaminergic neurons. Dopamine neurons discharge wide impulses (between 1.5 msec and > 2.0 msec duration with 100 Hz high-pass filter) at low frequency (usually 0–6 impulses/sec), as confirmed by optogenetic identification,^26^ and contrasting with all other neurons in the area.
  i. Conduct neuronal tests in both choice and no-choice trials. While choice trials implement the decision process conceptualized by economic choice theory, no-choice trials serve to reduce interference from the unchosen option. For no-choice trials, set the amount of both liquids of the not-to-be-chosen option to zero, which after a few repetitions lets the animal choose the non-zero option (Figure 2 step f).
  j. On every recording day, withdraw the electrode assembly and return the animal to its home cage. Give it some extra treat (e.g., banana, marshmellow), in particular when it cooperated well.

**CRITICAL:** Identification of neuron type by discharge characteristics is crucial for obtaining recordings from functionally or anatomically well-defined neuron types in heterogeneous brain structures.

***Note:*** Collect neuronal data during most working days of each week to provide similar conditions required for valid data comparisons and population analyses. A possible non-recording exception is Monday on which some animals perform less reliably because of weekend distractions.

**Figure 4:**
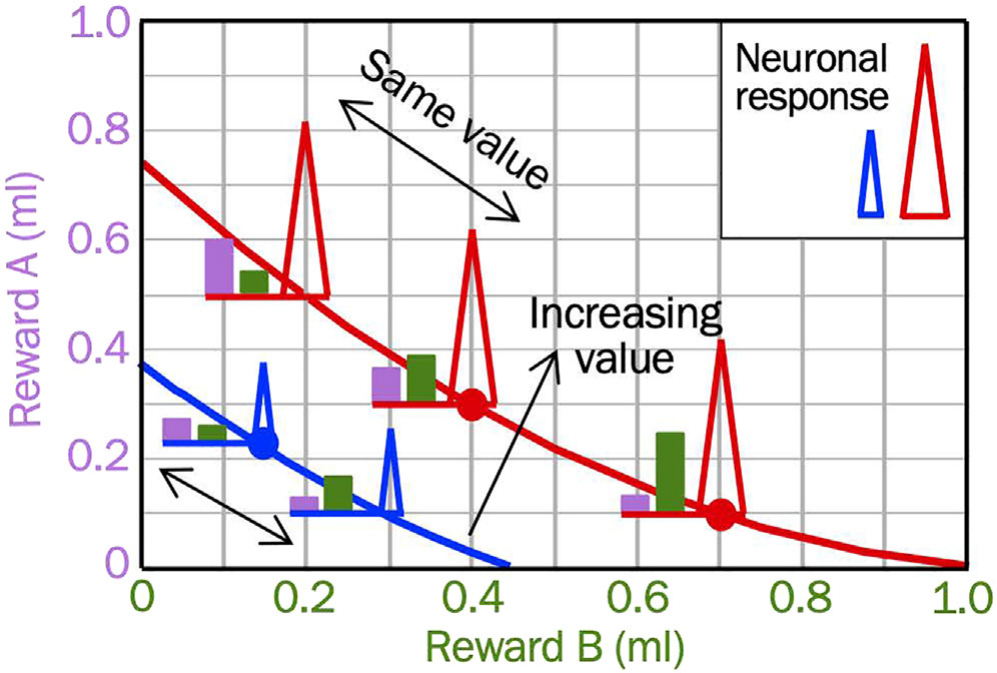
Neuronal response scheme for two-component choice options Indistinguishable neuronal response along same indifference curve (IC) on which all options have same subjective value (blue, red) despite varying option composition (pink, green), but different responses across ICs with different value. Height of small pink and green bars indicate respective amounts of Rewards A and B. Adapted from our earlier study.^4^
  b. Use adequate statistics.
    i. Use triple linear regression on physical reward amount for assessing significance of response change across ICs (one regressor for each component, one interaction regressor). Test correlation of neuronal responses with IC slope with the component regressors and IC curvature with the interaction regressor.
    ii. Use Spearman rank-correlation to confirm monotonic ordinal change across ICs.
    iii. Use two-factor Anova for response similarity along ICs: insignificant change along IC in Anova with significant across-IC factor.
    iv. Use adequate regressions to analyze neuronal responses during choice according to various forms of chosen value (absolute or relative chosen or unchosen value) (given the similar composite visual stimuli, the two choice options cannot be identified unequivocally by the animal, which precludes analysis in terms of object value).
37. Run an initial analysis to test whether the reward settings are suitable for revealing neuronal responses that follow the IC response scheme.
  a. Inspect the neuronal data every day, ideally with histograms and rasters using prepared analysis software.
  b. Search for statistically significant responses to the task events (stimuli, choice, reward delivery; Figure 1 step 2).
  c. Fine-tune the task parameters including reward amounts until neuronal responses follow the response scheme (Figure 4).
38. Finalize data collection and analysis.
  a. Once the task details have been finalized, record neurons in trial numbers that are sufficient for statistically assessing response significance (see above).
  b. Collect similar trial numbers with each neuron to have adequate data for comparing responses between neurons.
  c. Collect similar neuron numbers from at least two monkeys for reproducibility across animals. Otherwise extend recordings to a third or even a fourth monkey.
  d. Aim for a minimum of 50–100 neurons per monkey that show the characteristics specified by the task design (Figure 4), and a total of 100–200 neurons for the whole study.

***Note:*** Data variability is only tested across multiple neurons, not across monkeys. The low number of monkeys allows only data confirmation across animals and precludes specific comparisons or statistics. This procedure is justified by ethical considerations about minimal necessary animal experimentation.

35. Identify task-related neurophysiological responses.
  a. Consider four relevant task epochs.
    i. Visual stimuli predicting the options.
    ii. Go signal for making the choice.
    iii. Choice itself.
    iv. Reward delivery (separately for Liquids A and B).
  b. Count the number of neuronal impulses in each task epoch and in a pre-trial control epoch.
  c. Identify task-related responses with the paired Wilcoxon test for individual comparisons (task epoch vs. pre-trial control epoch) or the Kruskal-Wallis test or one-way Anova (all five epochs at once).
36. Test whether task-related neuronal responses follow the scheme of two-dimensional ICs.
  a. Establish a neuronal response scheme (Figure 4) that implements the characteristics of an IC map (Figure 1 step 5).
    i. Neuronal response increase or decrease across ICs (high > low).
    ii. Similarity along same ICs.

39. Use neuronal population responses to construct neuronal ICs.
  a. Analyze only neuronal responses that vary significantly across ICs but not along ICs.
  b. Obtain a normalized count for each response to a single option component for each neuron by calculating a z-score (subtraction from mean neuronal activity during pre-trial control period and division by standard deviation of that activity).
  c. Obtain a z-scored neuronal population count for each option by averaging all z-scored individual neuronal responses to that option. Thus, neurons with significant changes in > 1 task epoch may contribute multiple responses to the population average.
  d. Plot the tested options at the actual x-coordinate of component B and the estimated y-coordinate for component A derived from a neuronal regression interaction coefficient. Then fit hyperbolically neuronal population ICs to x-y points of similar neuronal response strength irrespective of behavioral ICs.
  e. Determine whether the neuronal population ICs fit well into the 95% confidence intervals of the hyperbolically fitted behavioral ICs to indicate precision of neuronal coding.

***Note:*** Analyze all behavioral and neuronal data separately for each monkey, thus respecting the individual subjectivity of reward value.

40. Use machine learning tools to predict behavioral choices from neuronal responses.^27^
  a. Use a linear support vector machine (SVM) to decode neuronal responses according to two ICs, using svmtrain and svmclassify procedures from Matlab.
    ***Note:*** The SVM decoder would find the optimal linear hyperplane for the best separation between two neuronal response populations relative to lower vs. higher ICs.
  b. Use linear discriminant analysis (LDA) to decode neuronal responses between and along ICs. The LDA decoder would find the axes (linear discriminants) for best separation between lower and higher ICs (first discriminant) and positions along individual ICs (second discriminant).
  c. To run either decoder, use 10 trials per neuron for each of two ICs (total of 20 trials). Inclusion of 15–20 trials per group does not seem to provide significantly better decoding rates but reduces the number of useable neurons.
  d. With neurons recorded for > 10 trials per IC, select randomly 10 trials for each IC.
  e. Implement the SVM/LDA decoder.
    i. Use a leave-one-out cross-validation method in which one of the 20 trials is removed and train the SVM/LDA decoder on the remaining 19 trials.
    ii. Then use the SVM/LDA decoder to assess whether it accurately detects the IC of the left-out trial.
    iii. Repeat this procedure 20 times, every time leaving out another one of the 20 trials.
    iv. The 20 repetitions result in percentage of accurate decoding (% out of n = 20).
    v. The final percentage estimate of accurate decoding results from averaging 150 iterations.
  f. To distinguish from chance decoding, randomly shuffle the assignment of neuronal responses to the tested ICs, which should result in an accuracy of 50% correct. Significant non-shuffled decoding would be expressed as statistically significant difference against the shuffled data (Wilcoxon test).
  g. For visualization with LDA, select randomly 10 trials from all neurons tested with two options located on lowest and highest IC. Attribute the first linear discriminant to separation between options on two different ICs, and the second linear discriminant to separation of options along same ICs.
  h. Check whether the accuracy of the prediction increases monotonically with the number of neurons included in the decoder, which indicates valid choice prediction.
  i. Check the number of neurons required for prediction accuracy of > 80%. Lower number of neurons indicates sparser coding.
    ***Note:*** The decoder approach models the influence of a neuron on postsynaptic neurons whose activity ultimately leads to the choice.

41. Test reward-specific satiety as an example of value change that should be manifested in value-coding reward neurons.
  a. Animals become naturally sated during on-going daily task performance.
  b. Study satiety from on-going consumption rather than from more artificial bolus injection.
  c. Select two rewards for which the animal will become sated with different speed and degree. Therefore, reward-specific satiety will be relative, not absolute.
  d. Satiety tests with two differentially sated rewards help to distinguish against general satiety that is not limited to value reduction and likely also reflects fatigue and loss of arousal and attention.
  e. As ICs reflect reward-specific value, ICs would change when the relative reward value is affected by satiety. Therefore, an IC map offers a diagnostic tool for relative reward-specific satiety. The altered IC map should be reflected in altered neuronal responses.

**CRITICAL:** Record from the same neuron before and after satiety.

## Expected outcomes

### Specific outcomes: Human choices

**Figure 5:**
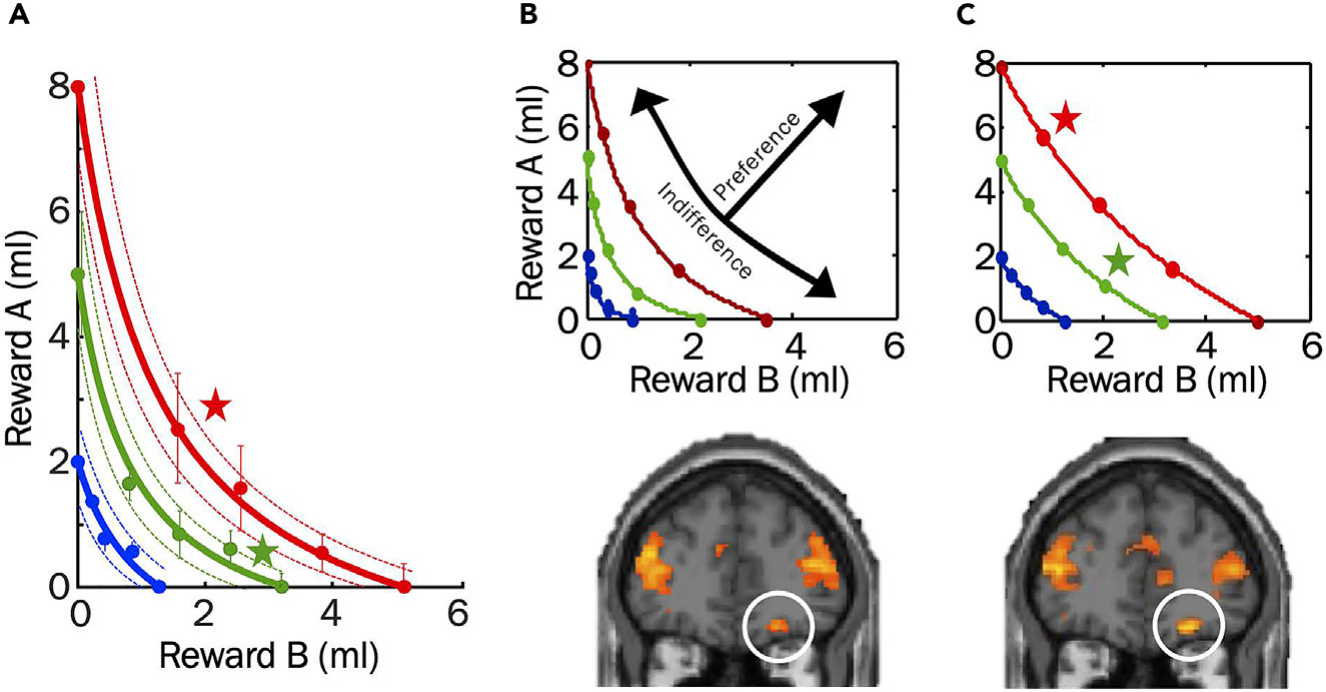
Expected behavioral and neural outcomes with human participants (A) Typical convex ICs from a human participant (hyperbolic fits to IPs). Dots show IPs, dotted lines show 95% confidence intervals of the fits. Stars show partial physical non-dominance. Adapted from our earlier study.^2^ (B) fMRI neuroimaging responses in orbitofrontal cortex (OFC; circle) follow two-dimensional indifference curves (ICs). Top: Test schematic; different BOLD signals across ICs (increasing value with increasing ICs) but similar signals within ICs (same value; arrows). Bottom: BOLD responses in OFC following two-dimensional ICs (map threshold p < 0.005, extent threshold ≥ 10 voxels): increase across ICs but no difference along ICs. (C) Higher OFC responses to more valued but physically partially dominated options on different ICs. Top: ICs. Bottom: stronger OFC responses to options on higher ICs compared to options in lower ICs. Map threshold p < 0.005, extent threshold ≥ 10 voxels. (B) and (C) adapted from our earlier study.^1^

Psychophysical assessment in humans using sugary and fatty liquids demonstrates the feasibility of experimentally measuring ICs for two-component options according to Revealed Preference Theory.^2^ In Figure 5A, all options on the same IC have the same subjective value and are equally preferred to each other, and options on higher ICs have higher value than, and are preferred to, options on lower ICs (options on red IC preferred to options on green IC, and options on green IC preferred to options on blue IC), despite their different reward amount composition. The preference relationship holds even when one reward component of the higher IC is lower than a reward component on a lower IC (partial physical non-dominance; stars). The scalar BDM bids replicate the value relationships expressed in the ICs: similar bids for options on same IC, but higher bids for options on higher ICs. SVM and LDA decoders confirm the orderly value representation by ICs. These human choices indicate scalar subjective value and scalar preferences for multi-component choice options.

### Specific outcomes: Human fMRI neuroimaging

Stimulus-induced fMRI neuroimaging responses in human OFC follow the two-dimensional IC graph: variation across ICs (high > low), and similarity along same ICs (Figure 5B).^1^ The stronger responses to options on higher ICs hold with different option composition and, amazingly, even when one of the components of the higher-IC option is smaller than in the lower-IC option (partial physical non-dominance) (Figure 5C; stars in top panel). fMRI responses to stimuli for individual options correlate with BDM bids during the bidding phase in ventromedial prefrontal cortex (vmPFC). These human neuroimaging responses indicate scalar subjective value signals for multi-component choice options.

### Specific outcomes: Monkey choices

**Figure 6:**
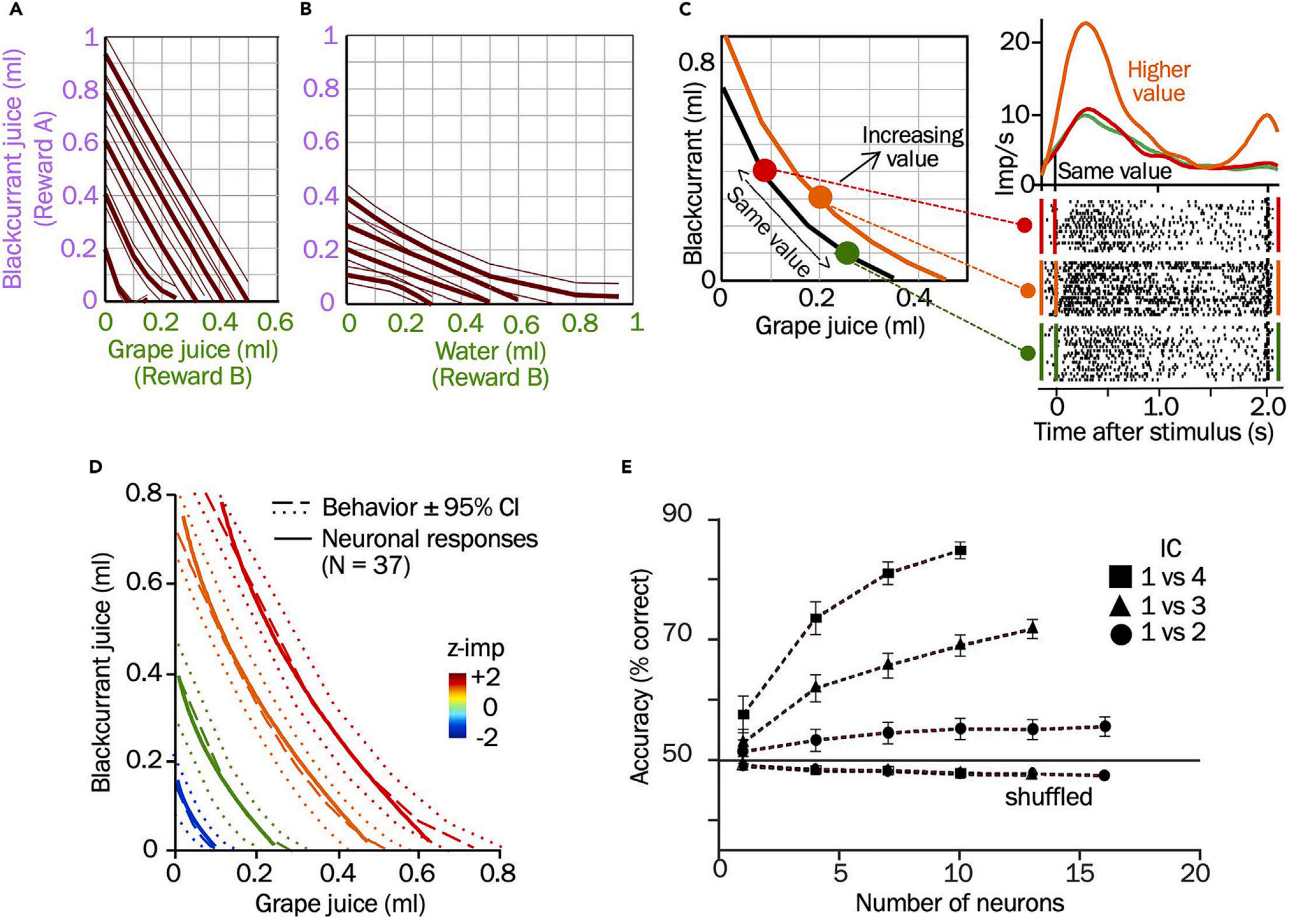
Expected behavioral and neurophysiological outcomes with monkeys (A) Five indifference curves (ICs) for two-component options containing blackcurrant juice and grape juice. Thin lines indicate 95% confidence intervals. (B) Four ICs for two-component options containing blackcurrant juice and water. (C) Responses in an orbitofrontal cortex (OFC) neuron that follow the IC scheme: same response to options on same IC (red and green) but stronger response to option on higher IC (orange). Smoothed peri-stimulus histograms (density functions) above raster displays of neuronal discharges. (D) Neuronal ICs in OFC neuronal population (22 neurons with 37 responses in any task epoch). Neuronal ICs (solid lines) are estimated from positively coding, non-simultaneously recorded neurons and align similar neuronal responses to different options irrespective of their behavioral IC. Behavioral ICs (dotted lines) are plotted from hyperbolic fits to choice indifference points (± 95% confidence intervals; z-imp/s: response strength). (E) Choice prediction by OFC neurons. In a support vector machine (SVM) decoder, increasing numbers of neurons predict choices between options on different ICs with increasing accuracy. (A) to (E) adapted from our earlier study.^4^

Extensive behavioral tests in tens of thousands of trials show ICs that are specific for particular option compositions (Figures 6A and 6B). Similar to the choices in the human participants, all options on their common IC have same subjective value despite different reward amount composition, and all higher-IC options have higher value than all lower-IC options even with lower amount of one reward component of the higher-IC option. Choice consistency is suggested by out-of-sample and transitivity tests.^3^ These data demonstrate systematic and meaningful choices of two-component options that indicate how scalar subjective value and scalar preferences emerge from multi-component choice options.

### Specific outcomes: Monkey neurophysiological recordings

The responses of individual OFC neurons in monkeys correspond closely to the characteristics of the behavioral ICs representing scalar subjective reward value and preferences of multi-component choice options (Figure 6C).^4^ The OFC population responses fit inside the 95% behavioral confidence intervals (CI; Figure 6D). Linear decoders show that neuronal responses predict the behavioral choices with 80% accuracy with as few as 10 neurons (Figure 6E). Thus, OFC neurons show scalar subjective value signals for multi-component choice options.

**Figure 7:**
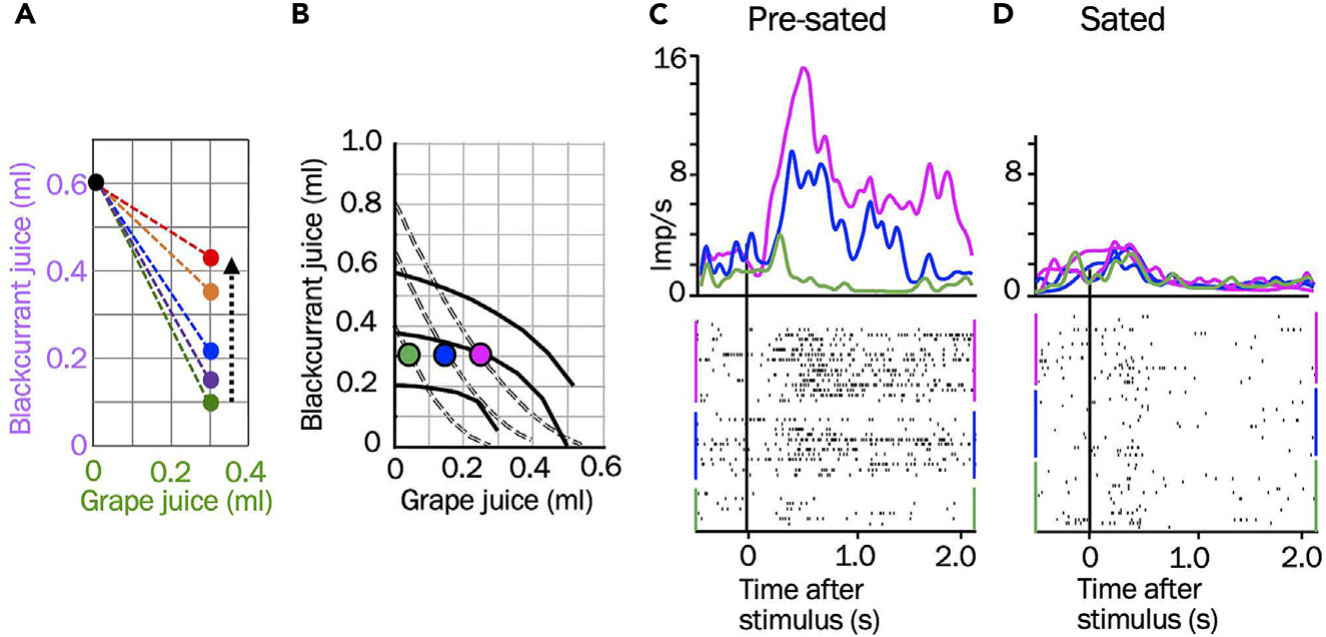
Relative, reward-specific, satiety-induced, value change in monkeys (A) Gradually flattening choice indifference slopes between two options with increasing consumption of both rewards (arrow), indicating satiety-induced gradual value loss of grape juice relative to blackcurrant juice. (B) Change of indifference curves (IC) following relative reward-specific satiety. Dotted ICs: before satiety. Solid ICs: during satiety: the flat slope reflects absence of value gain despite increasing grape juice amount. After the consumption-induced IC slope and curvature change (from convex to concave), the three physically unchanged options are now on or close to the same intermediate IC, indicating similar subjective value among them as a result of satiety for grape juice (the animal fails to give up much blackcurrant juice for the now low-valued grape juice). (C) Before satiety: monotonic response increase in single orbitofrontal cortex (OFC) neuron across three ICs (dotted lines in panel B) with increasing grape juice (constant blackcurrant juice). (D) Lack of neuronal response increase for increasing amounts of grape juice on which the animal is sated. (A) to (D) adapted from our earlier study.^5^

The neuronal responses follow intuitive value changes induced by reward-specific satiety (Figure 7).^5^ During on-going task performance, the animal gets sated in a reward-specific manner: it gives up progressively less of the less sated reward to gain one unit of the more sated reward, thus flattening the IC slope (Figure 7A) and changing the initially convex ICs into concave (Figure 7B; dotted grey vs. solid black lines). Whereas neuronal responses increase across increasing ICs before satiety (Figure 7C; from green to red), they collapse and fail to increase during satiety (Figure 7D). Thus, the decaying neuronal responses match the satiety-reflecting IC changes and confirm the neuronal value coding of two-component choice options.

## Limitations

Design issues: behavioral experiments that are designed ad-hoc without relation to specific theories or are poorly controlled may result in data that are difficult to interpret. Thus, theories proven by hundreds of conceptual challenges and empirical tests over many decades provide solid guidance. However, the restrictions of such theories need to be critically evaluated, as too much control and abstraction may prevent unexpected discoveries.

Theory-driven research is limited by the availability of the necessary theory. The employed psychological learning theories and economic decision theories are well developed but do not allow much spontaneous wild-type tests. By contrast, spontaneous and imaginative tests can produce novel data but may result in uninterpretable data without the possibility of forming coherent theories or completing existing theories.

Behavior restrained by laboratory situations has advantages (controlled variables for regressions) but also disadvantages (non-natural situation). The opposite end is made up of primarily observational studies in more natural situations, although often inadvertent intercorrelations between regressors may result in interpretational issues.

Neurophysiology on single neurons or small sets of neurons provides precise temporal and spatial resolution but cannot investigate oscillations, synchrony and network properties. Electrode arrays (e.g., Utah Array) are better suited for these purposes but make testing more stereotyped and thus reduce individualized on-line testing of neurons according to their observed task relationships.

The study of brain signals underlying the tested behaviors provides important information about the neuronal implementation of crucial behavioral variables, such as reward value and preference. However, such studies do not explain how the signals lead to behavior, which would require to demonstrate functional causality, and specifically sufficiency. Sufficiency can now be demonstrated by optogenetic stimulation. By contrast, necessity studied by lesions and functional interference, although causal in a logical sense, does not allow to assess how a given brain structure or process drives or ‘causes’ behavior.

## Troubleshooting

### Problem 1

Protocol step 4: An IC may not have a downward and rightward slope, which may indicate a more complicated substitution of one option component by the other component. For example, an upward slope indicates that more Liquid A (y-axis) is required for consuming more Liquid B (x-axis), which may indicate that Liquid B has negative value: increasing amounts of Liquid A are necessary to compensate for the increasing aversiveness of larger amounts of Liquid B (like sweet marmalade for bitter yoghourt).

### Potential solution

Change Liquid A until ICs show a downward and rightward slope.

### Problem 2

Protocol step 13: BDM isolines in humans do not fall within the 95% confidence intervals of ICs. The problem may be due to the somewhat abstract nature of the BDM compared to the more intuitive binary choice. It is well-known that correct BDM performance is not always achievable in every participant.

### Potential solution

More testing of the particular participant presenting the problem, and replacing the participant if the problem persists.

### Problem 3

Protocol step 27: When choosing between two quantitative stimuli (Figure 2 steps c and d), a monkey does not prefer the higher reward amount in more than half the trials.

### Potential solution

The monkey may be uncomfortable in the primate chair in the laboratory, be insufficiently or too rapidly trained, or simply myopic. Therefore:

• Check all parts of the chair that might exert strong contact or pressure on the animal. A persistent touch of the chair by a head-fixed animal, even for only a few minutes, can result in pressure and impair local blood circulation, thus generating itching or hypoesthesia that makes the animal jittery. The slightest discomfort can impair a monkey’s performance.
• Check for signs of poor health, which may require inspection by a veterinarian, and check for signs of hunger and thirst, which can be easily remedied.
• If the problems persist, provide further training to address potentially insufficient experience in the laboratory. Also, reduce task demands, even below the level of the desired tests, to make the animal more comfortable. Then increase task demands very gradually again while keeping the animal’s performance above 75%–90% correct.
• If all negative factors can be ruled out, myopia may provide an explanation. To address that possibility, position the computer monitor closer to the animal.

### Problem 4

Protocol step 28: Monkeys’ ICs depend on direction of testing. ICs estimated by advancing the tested options in opposite directions may not overlap.^15^

### Potential solution

Such lack of overlap may occur particularly in unexperienced monkeys when the Variable Option advances from the Reference Options over longer distances towards and beyond the center of the x-y map. Address the problem by training for several more weeks or even months.

### Problem 5

Protocol steps 39, 40: Neuronal responses do not follow the IC scheme (Figures 6D and 6E).

### Potential solution

Go back to the reduced choice between two one-component options (Figure 2 step d). If this does not help, use the reduced no-choice option (Figure 2 step f) but with only one component in each option. Only advance to the full option set (Figure 2 step e) when the animal performs well in both reduced tasks. Make these steps gradually.

## Resource availability

### Lead contact

Further information and requests for resources and reagents should be directed to and will be fulfilled by the lead contact, Wolfram Schultz (wolfram.schultz@protonmail.com).

### Materials availability

This kind of study will not generate unique products.

## Data Availability

Data and code will be made available upon reasonable request to the lead contact.
